# Reproducibility warning: The curious case of polyethylene glycol 6000 and spheroid cell culture

**DOI:** 10.1371/journal.pone.0224002

**Published:** 2020-03-19

**Authors:** Simona Serrati, Chiara Martinelli, Antonio Palazzo, Rosa Maria Iacobazzi, Mara Perrone, Quy K. Ong, Zhi Luo, Ahmet Bekdemir, Giulia Pinto, Ornella Cavalleri, Annalisa Cutrignelli, Valentino Laquintana, Nunzio Denora, Francesco Stellacci, Silke Krol

**Affiliations:** 1 Nanotechnology Laboratory, IRCCS Istituto Tumori "Giovanni Paolo II", Bari, Italy; 2 IFOM-The FIRC Institute of Molecular Oncology, Milan, Italy; 3 Experimental Pharmacology Laboratory, IRCCS Istituto Tumori "Giovanni Paolo II", Bari, Italy; 4 Institute of Materials, Ecole Polytechnique Federale de Lausanne (EPFL), Lausanne, Switzerland; 5 Department of Physics, University of Genoa, Genoa, Italy; 6 Department of Pharmacy- Pharmaceutical Sciences, University of Bari "Aldo Moro", Bari, Italy; 7 Interfaculty Bioengineering Institute, Ecole Polytechnique Fédérale de Lausanne (EPFL), Lausanne, Switzerland; 8 Laboratory for personalized medicine, National Institute of Gastroenterology IRCCS "S. de Bellis" Research Hospital, Castellana Grotte, Bari, Italy; VIT University, INDIA

## Abstract

Reproducibility of results is essential for a well-designed and conducted experiment. Several reasons may originate failure in reproducing data, such as selective reporting, low statistical power, or poor analysis. In this study, we used PEG6000 samples from different distributors and tested their capability inducing spheroid formation upon surface coating. MALDI-MS, NMR, FTIR, and Triple SEC analysis of the different PEG60000s showed nearly identical physicochemical properties different, with only minor differences in mass and hydrodynamic radius, and AFM analysis showed no significant differences in the surface coatings obtained with the available PEG6000s. Despite these similarities, just one showed a highly reproducible formation of spheroids with different cell lines, such as HT-29, HeLa, Caco2, and PANC-1. Using the peculiar PEG6000 sample and a reference PEG6000 chosen amongst the others as control, we tested the effect of the cell/PEG interaction by incubating cells in the PEG solution prior to cell plating. These experiments indicate that the spheroid formation is due to direct interaction of the polymer with the cells rather than by interaction of cells with the coated surfaces. The experiments point out that for biological entities, such as cells or tissues, even very small differences in impurities or minimal variations in the starting product can have a very strong impact on the reproducibility of data.

## Introduction

One of the major concerns about the quality of research is the reproducibility of either the own data or also from other researchers, as Baker showed with the results from a survey answered by 1500 scientists [1]. Numerous reasons have been identified for the failure in reproducing data, such as selective reporting, low statistical power, or poor analysis. Especially in medicine, chemistry, and biology, the reproducibility of data is an issue.

In this study, where biology meets chemistry for medical applications, we identified another reason which hampers data reproducibility. We performed a surface coating by depositing polyethylene glycol with a molecular weight of 6000 Da from different distributors. The study was initially aimed develop an easy and cheap technology to produce cell spheroids. However, the results obtained motivated us to investigate in detail the issue of data reproducibility when biological entities, like cells, interact with same polymer obtained from different distributors. Experiments were carried out in two different laboratories by four different operators repeatedly to ascertain the reproducibility of the findings. We used a variety of cell lines to eliminate the possibility that the observations were due to specific cell-polymer interactions.

Three-dimensional (3D) tumor cell culture became an important tool as realistic test bed especially for nanoparticulated drug delivery, nanotoxicity as well as pharmaceutical drug, testing as it mimics more closely the physiological environment in a tumor in terms of accessibility, presence of extracellular matrix and intercellular communication, as compared to conventional monolayer (2D) cell culture [2]. Long-term incubation with nanoparticles (NPs) can often lead to aggregation of the NPs which then precipitate and accumulate on the cell surface in 2D cell culture. These aggregates are up-taken into cells by phagocytosis and can induce cell toxicity or deliver drugs in a very different way with respect to the toxicity or drug delivery by single NPs [3,4]. On the other hand, the presence of extracellular matrix and the assembly of several layers of cells make floating spheroids a good model for the development and optimization of efficient intratumoral nanoparticulated drug delivery [5,6] and for drug penetration and diffusion studies [7–10]. Moreover, it has been demonstrated that large spheroids (>200 μm in diameter) form the three different regions of a tumor, i.e. a proliferating periphery region, a viable but quiescent intermediate region and a necrotic core [7,11,12]. The spheroids recapitulate *in vivo* tumor-like development patterns of avascular tumor nodules, in terms of morphology and growth kinetic properties [13–15].

Different techniques are available to grow small tumour spheroids. One of the simplest method is the hanging drop method [15]. Others created cell-repellent surfaces by chemically modifying or microcontact printing the culture dish or glass with PEG or super hydrophobic molecules [16–18].

We employed a simple coating procedure of the culture dish with PEG6000 for 1 h at 37°C. Only for PEG6000 from Carlo Erba (C.E.), the cultivated cells for different cell lines formed compact spheroids of varying size. If the coating was carried out with PEG6000 from other distributors (Merck, Sigma-Aldrich (S.A.), Acros) we observed cells growing in 2D. In order to identify the difference in the chemical composition, all PEGs were analysed in detail their by NMR (nuclear magnetic resonance), FTIR (Fourier transform infrared spectroscopy), triple SEC (triple detection size exclusion chromatography), DSC (differential scanning calorimetry) and by MALDI-MS (matrix-assisted laser desorption/ionization mass spectroscopy). We found small differences in the molecular weight and viscosity. AFM (atomic force microscopy) experiments were performed to analyse PEG6000 coating of the culture dish surfaces. experiments performed by pre-incubating the cells with the polymer and, after washing, depositing them on uncovered culture dishes indicated that the cell-polymer interaction is the main reason for the difference in cell growth. These experiments point out the importance of precise description of the purchased product, in order to allow reproducibility.

## Materials and methods

### Cells

HT-29 (ATCC^®^ HTB-38^™^, LGC Standards S.r.l., Italy) cells were cultured in Modified McCoy's 5a Medium (Euroclone, Italy) while Caco-2 [Caco2] (ATCC^®^ HTB-37^™^,LGC Standards S.r.l., Italy) were kept in Eagle's Minimum Essential Medium, (Euroclone, Italy). Both cell lines originate from human epithelial colorectal adenocarcinoma. PANC-1 (ATCC^®^ CRL-1469^™^, LGC Standards S.r.l., Italy), a human pancreatic tumor cell line, was grown in Dulbecco's Modified Eagle's Medium (Euroclone Italy), while HeLa (ATCC^®^ CCL-2^™^, LGC Standards S.r.l., Italy), cells from cervical human adenocarcinoma were cultured in Eagle's Minimum Essential Medium (Euroclone Italy). For all cell lines the following components were added to the basic culture medium: 10% foetal bovine serum (FBS; Gibco, Thermo Fisher Scientific, Inc., Waltham, MA, USA), 1% glutamine (Euroclone, Italy), and 1% penicillin/streptomycin (Euroclone, Italy). Cells were cultured in an incubator at 37°C in an atmosphere containing 5% of CO_2_. Frequent mycobacteria tests are performed.

### Reagents

Polyethylene glycol with an average molecular weight of 6000 Da (PEG6000) was purchased from C.E. (cat. n°: A192280010; out of production), S.A. (cat. n°: 1546580), Merck Millipore (cat. n°: 8.07491), and C.E. as distributor for Acros organics (Cod. 192280010 Lot. A0398882). Polyethylene glycol 4000 (PEG4000) was purchased from Polichimica (Bologna, Italy, LOT no. 97001725).

### 3D cell culture

96-well cell culture plates (Costar, tissue culture treated; Corning, n°. 3596) were coated to obtain a cell-repellent surface by exposing the wells for 1 hour at 37°C to 200 μL of 3% (w/v) PEG6000, in Milli-Q water filtered with 0.22 μm filters. Then the PEG solution was removed and 12x10^4^ cells were seeded in a final volume of 150 μL for each well. Cells were cultured for 24 up to 96 hours at 37°C, 5% CO_2._ For each of the four PEG6000 compounds, experiments were reproduced by four different operators in two independent laboratories.

For the experiments with PEG4000, we coated the surface with two concentration, 3% and 5% (w/v) solution. The plate was incubated with PEG4000 alone, or PEG4000 mixed with PEG6000 in a 1:1; 1:5 or 1:10 ratio. 1:5 is the ratio in height calculated from the two peaks observed in the MALDI spectrum for PEG6000 fromC.E..

Additionally, HeLa and HT-29 cells were incubated for 5 mins in a 3% PEG6000 solution from C.E. and S.A. For the experiment with short incubation, 12x10^4^ cells were deposited in an untreated 96-well plate and grown as described before for 48 h.

Cells were visualized by light microscopy using an OLYMPUS CKX41 microscope with a 10X/0.25 PHP objective.

### Nuclear Magnetic Resonance (NMR)

^1^H NMR was performed with a Bruker AV-400 MHz after dissolving the PEG6000 in D_2_O. The NMR data were analyzed with MestreNova.

### Attenuated Total Reflectance-Fourier Transform Infrared (ATR-FTIR) spectroscopy

The ATR-FTIR spectra were recorded using the IR spectrometer Thermo Fischer Scientific Nicolet 6700. 2 mg of the PEG samples were placed on the diamond crystal. The ATR measurement mode was used. The explored spectral range was 4000–600 cm^-1^. 64 scans were accumulated with a resolution of 4 cm^-1^ for each measurement. The spectra were processed using OMNIC spectra software to subtract the background and adjust baseline.

### Matrix Assisted Laser Desorption Ionization-Time of Flight Mass Spectrometry (MALDI-TOF-MS)

For MALDI-TOF, the PEG samples were dissolved in methanol to prepare a 5 mg/mL solution. α-Cyano-4-hydroxycinnamic acid (CHCA) was used as the matrix and was dissolved in methanol at a concentration of 10 mg/mL. 10 μL of PEG solution was then carefully mixed with 10 μL matrix solution in an Eppendorf tube. 2 μL of the mixture solution was spotted onto the Bruker stainless steel target and completely dried under vacuum. MALDI-TOF mass spectra were obtained using a Bruker AutoFlex spectrometer. The mass spectra were measured with positive ionization and linear mode with laser power at 25% attenuation. Spectra were processed and plotted using FlexAnalysis software.

### Mass Spectrometry (Q-TOF-MS)

Mass spectrometry measurements were carried out on Agilent 6530 accurate mass Q-TOF. PEG samples were dissolved in methanol:water 50:50 v/v and the solutions (0.1 μM c.a.) were filtered through PTFE membranes (0.2 μm, Agilent, CA, USA). Then, the filtrates were directly analysed by Q-TOF-MS by continuous infusion at a flowrate of 10 μl/min. Mass spectra were achieved in positive (ESI+) electrospray ionization mode. The spectra were processed using MassHunter B0600 software.

### Triple Size Exclusion Chromatography (SEC)

The size exclusion chromatography was performed using the OMNISEC Triple Detection system produced by Malvern Panalytical, equipped with refractive index (RI), viscosimeter, and right-angle light scattering (RALS). The eluent was 0.1 M NaNO_3_ solution, flow rate 0.6 mL/min, column set G2500PWXL + G3000PWXL and the dn/dc applied was 0.14. The samples were dissolved in a small volume of the eluent and 100 μL of this solution were injected.

### Differential Scanning Calorimetry (DSC)

DSC curves were obtained with a Mettler Toledo DSC 822e Stare 202 System (Mettler Toledo, Switzerland) equipped with an automatic thermal analysis program, as reported elsewhere [19]. Samples were heated from -20°C to 80°C, at a rate of 5°C/min, under a nitrogen flow of 50 mL/min.

### Atomic Force Microscopy (AFM)

Samples for AFM analysis were prepared by dissolving PEG in Milli-Q water to a concentration of 3% (w/v). A drop of PEG solution was placed on a polystyrene Petri dish (Iwaki, 1000–035, non-treated) for 1 h at 37°C. Then the solution was removed, and Milli-Q water was added. Hydrated samples were then mounted into the AFM liquid cell. AFM measurements were carried out using a Multimode/Nanoscope V system (Bruker). AFM images were acquired in contact mode in liquid (MilliQ water) using commercial Si3N4 cantilevers (DNP-10 Bruker, k = 0.24 N/m). For some samples PEG solution was not removed and AFM measurements were performed in PEG solution. No significant differences were observed between images acquired in Milli-Q water or PEG solution. Data were analyzed with Gwyddion software.

## Results

### Spheroid culture

**Fig 1:**
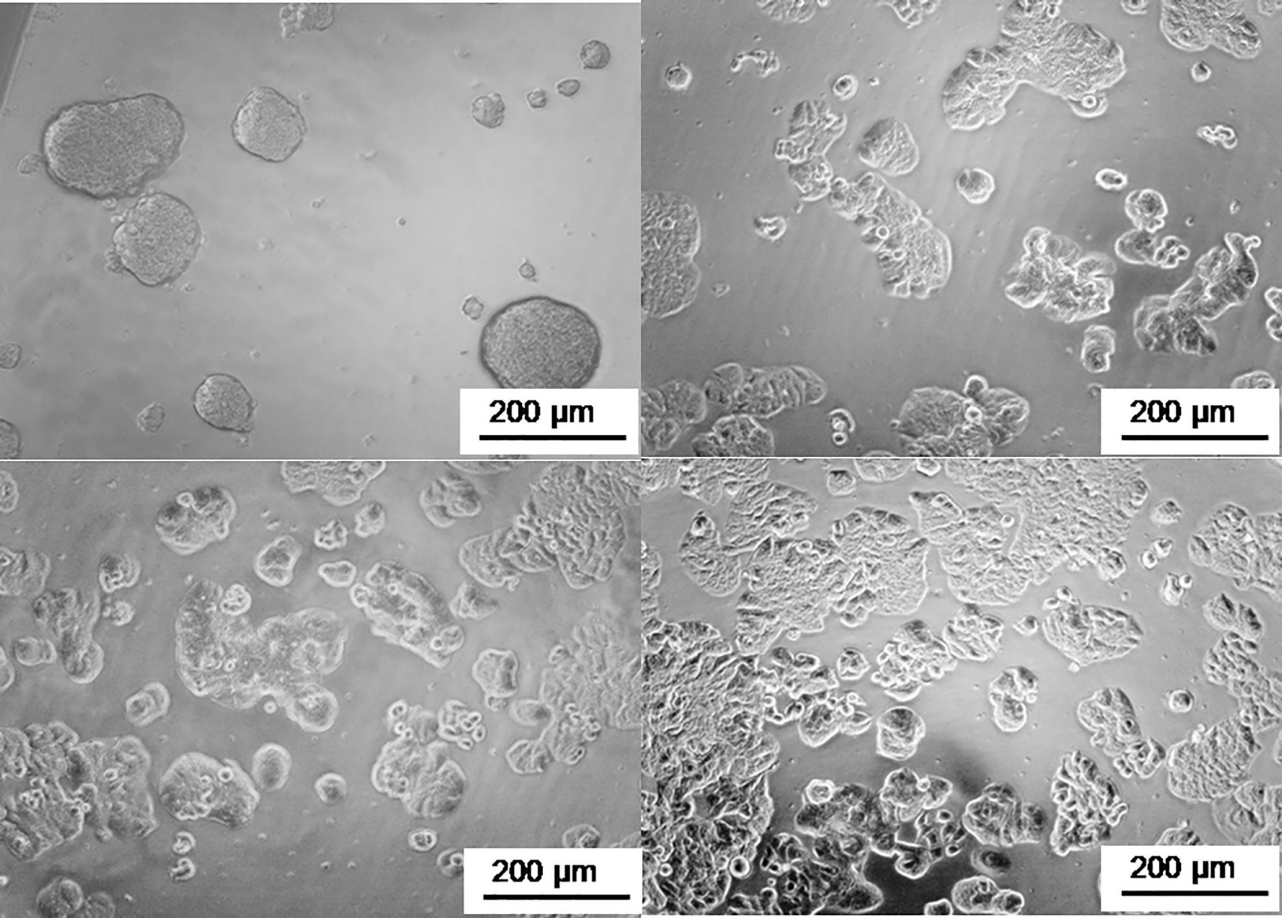
Microscopic images of HT29 cells on surfaces (1 h; 37°C) treated with PEG6000 from A) C.E.; B) Acros; C) Merck; and D) S.A.. The images were recorded 48 h after cell plating.

We prepared the surface coating of the 96-well dish with PEG6000 from different distributors using always the same concentration (3%) and incubation conditions (37°C, 1 h). As shown in Fig 1, only PEG6000 from C.E. provided a cell-repellent surface that allowed to obtain tight and well-defined floating 3D spheroids (Fig 1A) in the solution rather than a 2D cell layer.

**Fig 2:**
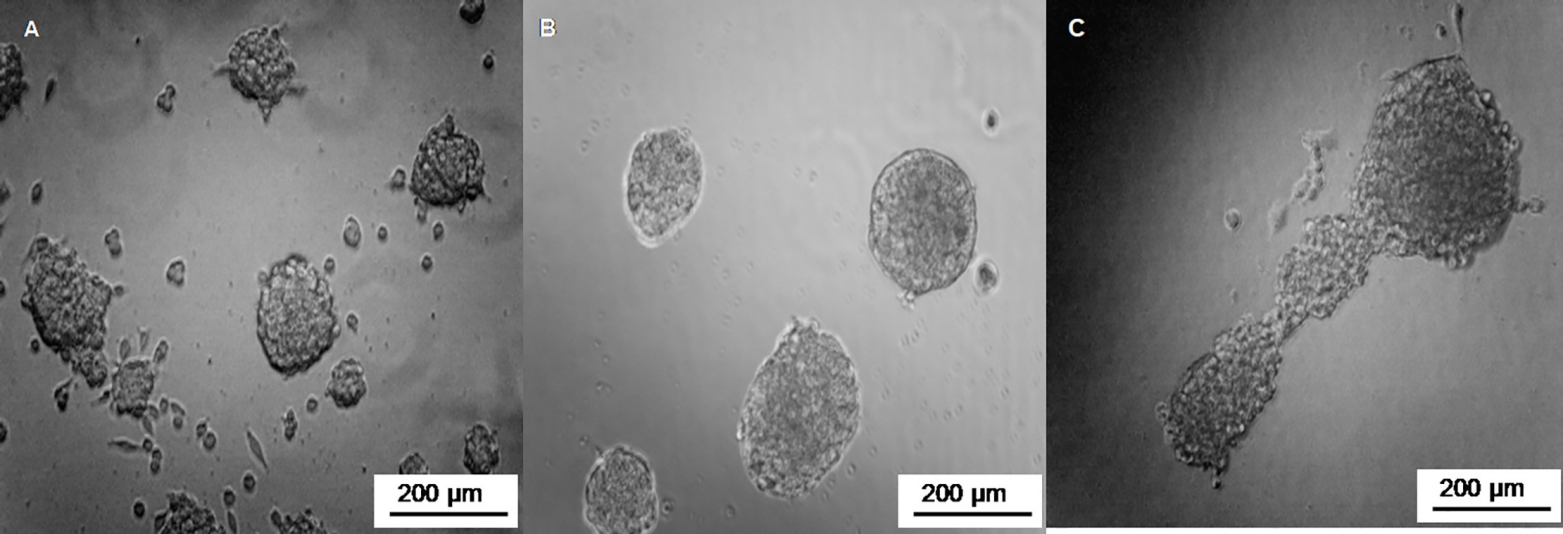
Micrographs of (A) HeLa, (B) PANC-1; and (C) Caco-2 cells imaged 72 h after seeding on PEG60000 (C.E.) pre-treated surfaces.

In order to exclude that it was an exceptional result due to properties of the cell line HT29, we performed the same experiments with different cell lines, such as Caco-2, PANC-1, or HeLa. As depicted in Fig 2, all the tested cell lines showed the same spheroid formation when PEG6000 from C.E. was used to coat the well plates.

The difference in cell behaviour on surfaces pre-treated with PEG6000 from different distributors led us to further investigate the physicochemical properties of the purchased PEG6000.

### Physicochemical characterization of the PEG6000

**Fig 3:**
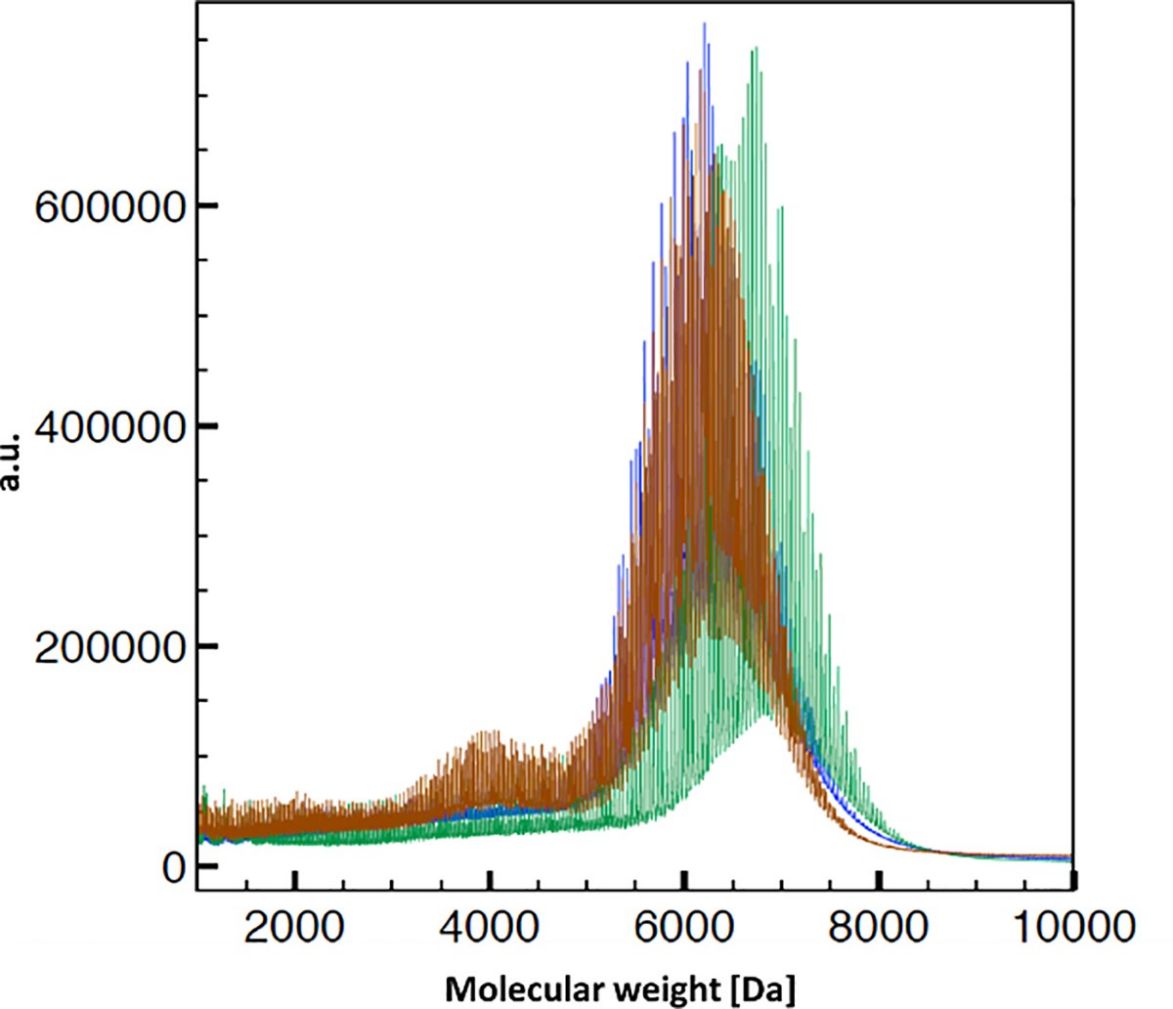
MALDI measurements to determine the mass of PEG 6000 produced by S.A. (green), Merck (blue), C.E. (red).

Firstly, we measured the mass of the different PEGs either by MALDI-MS, triple SEC, or ESI-TOF-MS. MALDI spectra of PEG6000 from different distributors showed small differences (Fig 3). While the average mass for PEG6000 from C.E. and Merck was around 6000 Da, as expected, the average mass for PEG from S.A. and Polichimica was closer to 7000 Da. The only difference we detected in the mass spectrum of PEG from C.E was an additional peak at 4000 Da with a relative ratio of 1:5 to PEG6000 (calculated by peak height).

The mass spectra in positive (ESI^+^) electrospray ionization mode (S3 Fig) showed multiple charged ions in the range of 400–900 Da for the PEG samples at lower m/z values to molecular weight, with a pattern of signals and with multiple isotopic peaks for each oligomer unit. There were no significant differences between the PEG6000 from C.E., Merck, S.A., and Acros. The average molecular weight of 5940 Da was calculated using the m/z value equal to 685.5 ([M+ 9Na^+^]^+^). The charge (z = 9) was calculated from the difference between the carbon isotopic peak which was 0.11. The additional molecular weight at 4000 Da cannot be distinguished from the signal of PEG6000 by this technique.

**Fig 4:**
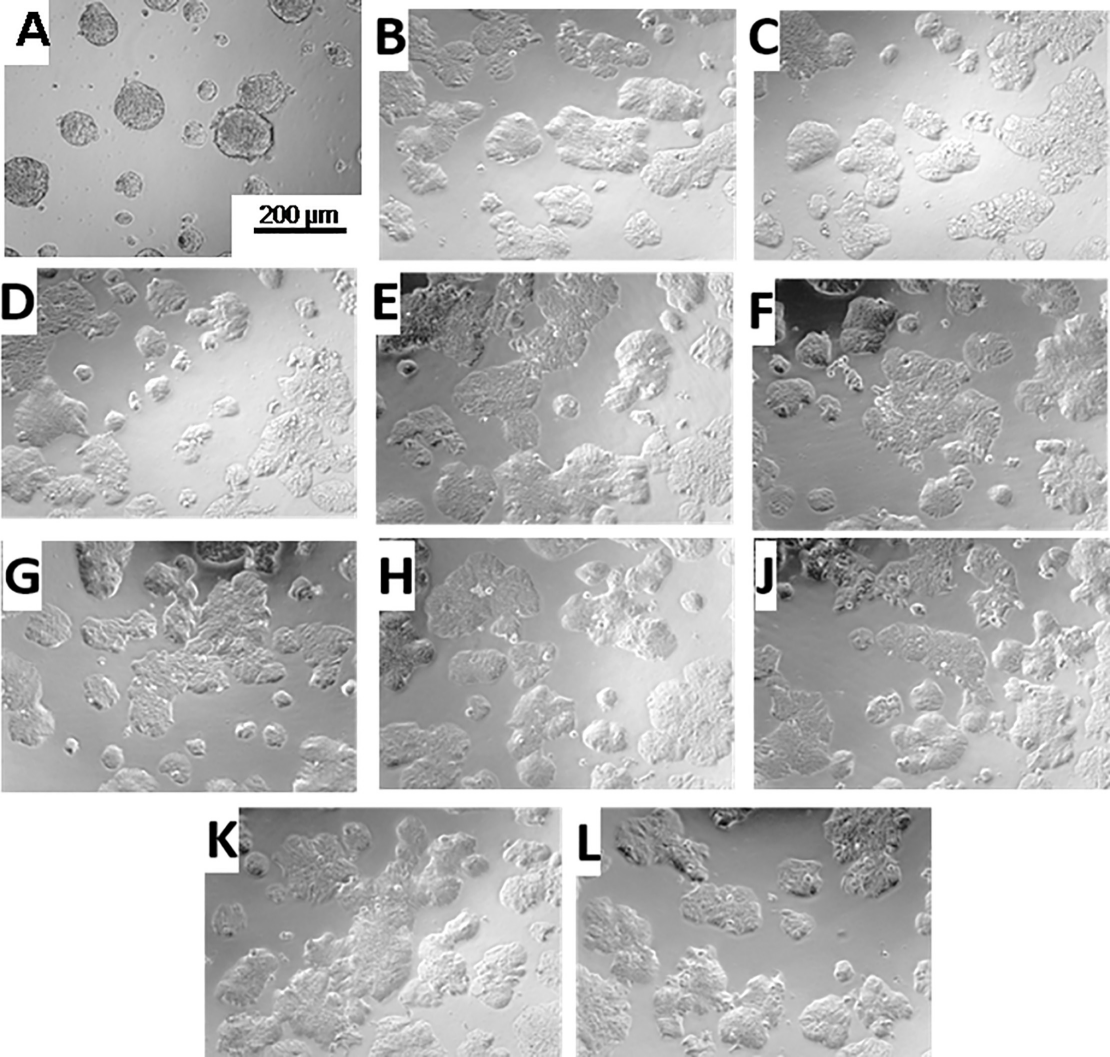
HT29 imaged by transmission light microscopy 96 h after plating in a 96 well plate treated for 1 h at 37°C with (A) PEG6000 from C.E., (B) PEG4000 (4000), (C) PEG6000 from S.A. (D) a mixture of 4000/S.A. 1/5 PRE diluted, (E) a mixture of 4000/S.A. 1/5, (F) mixture of 4000/S.A. 1/10, (G) PEG6000 from MERCK, (H) 4000/MERCK 1/5 pre-diluted, (J) 4000/MERCK 1/5, (K) 4000/MERCK 1/10, and (L) as control HT29 cells on an untreated surface. The scale bar in a) is valid for all images.

We performed FTIR and NMR measurements to determine the nature of the lower molecular weight peak. As it can be seen in S1 Fig, the spectra in FTIR were substantially similar for the 3 tested PEG solutions. The measurements in ^1^H-NMR (S2 Fig) confirmed that the chemical identity of the molecules was the same. From the NMR and FTIR measurements, it was clear that the second mass peak was PEG4000 in the C.E. PEG. In order to understand if this lower weight PEG was responsible for the cell-repellent properties, we coated the surface with PEG4000 (Polichimica) alone, using different concentrations (3%, 5%), or in combination with different ratios of PEG6000 (from S.A. or Merck): PEG4000 (1:1; 1:5; 1:10), and using different mixing procedures (Fig 4).

As it can be seen in the micrographs in Fig 4, when PEG4000 (Polichimica) was added in different ratios to PEG6000 from other distributors (in order to simulate the PEG composition of PEG6000 from C.E.), cells still grew in 2D and not as spheroids.

Cells are known to be responsive to micro- and nano-structured surfaces (e.g. [20]). In order to understand if a pattern of microstructures was formed on the culture dish surface, two experiments were performed: i) triple size exclusion chromatography (SEC) to determine if the PEG solution contained polymer molecules as stable random coils and ii) atomic force microscopy (AFM) imaging to understand if these structures were deposited on the surface.

Triple SEC combines measurements of refractive index, viscosity, and right-angle light scattering (RALS), in order to determine the molecular weight and the viscosity to get information on the structure of the polymers. The results are summarized in Table 1 and the measurements are shown in S4 Fig.

**Table 1:** Triple SEC measurement with PEG6000 from S.A., C.E., and Merck.

Sample	Mn (Da)	Mw (Da)	Mw/Mn	η (dl/g)	Rh (nm)	Recovery (%)
S.A.	6297±30	6342±40	1.008±0.002	0.1695±0.0007	2.565±0.007	94.64±0.14
C.E.	5843±30	5954±30	1.019±0.011	0.163±0.002	2.475±0.002	95.35±0.04
Merck	6019±2	6088±25	1.012±0.004	0.166	2.51	95.58±0.06

Number of measurements: n = 2; Mw: weight-average molar mass, Mn: number-average molar mass; Mw/Mn: polydispersity; η: intrinsic viscosity; Rh: hydrodynamic radius.

The SEC measurements confirmed a slightly lower molecular weight of PEG6000 from C.E. and Merck, as already measured by MALDI. Moreover, PEG6000 from C.E. had also the lowest intrinsic viscosity and a smaller hydrodynamic radius, which could be indicative for a more compact, coiled structure. This was also supported by the DSC analysis (S5 Fig) which showed a slightly higher melting temperature of 64.4°C for PEG6000 from C.E., as compared to those for Merck, and S.A. of 63.7°C and 62.1°C, respectively. Also, these values are in good agreement with the viscosity and hydrodynamic radius determinations and the compactness of the structure. Acros was included to confirm that there were no differences for this PEG6000, which has a melting temperature of 62.6°C.

Next, we investigated if the small differences in hydrodynamic radius, measured in triple SEC and confirmed by calorimetry, had an influence on the surface coating of PEG on the cell culture dish. The hypothesis was that PEG6000 from C.E. could induce a complete cell-repellent surface coverage, while the PEG6000 from the other distributors might not attach to the plastic surface or induce polymer patches. Then the solution was replaced by water and the surface was imaged by atomic force microscopy (AFM) in contact mode.

**Fig 5:**
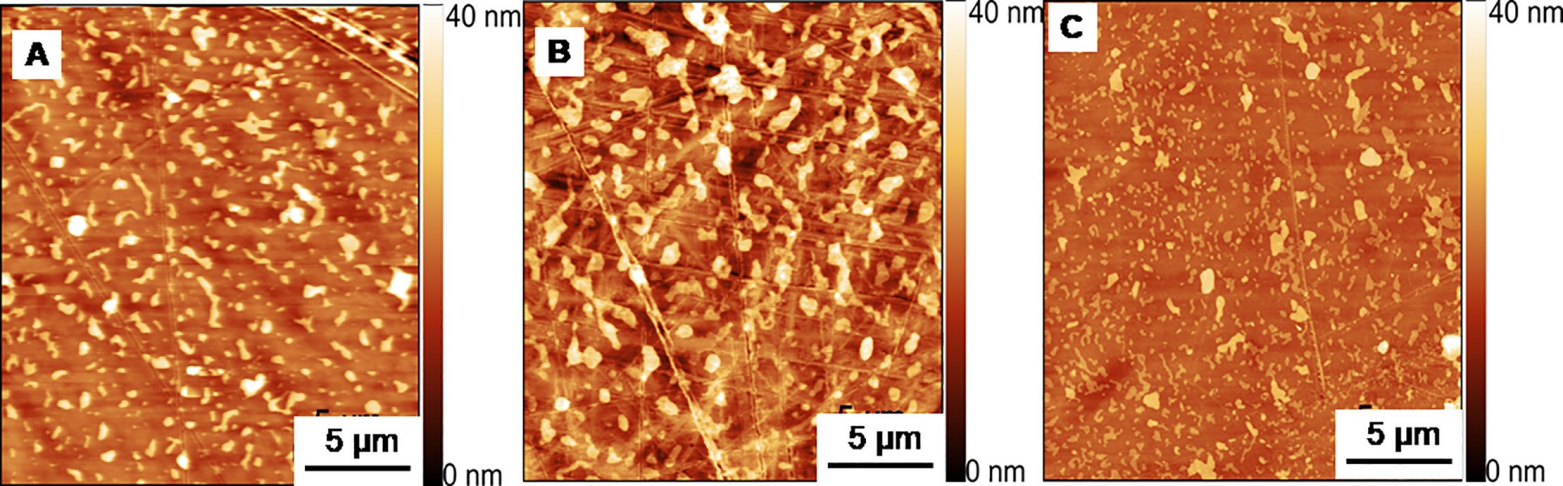
AFM micrograph of Petri dish surfaces incubated for 1 h with 3% PEG6000 from (A) C.E.; (B) Merck; and (C) S.A. The images were recorded after replacing the PEG solution with Milli-Q water. The scale bars indicate 5 μm.

AFM images in Fig 5 show some remarkable differences in surface pattern, island size, height or distance between the polymer islands. These differences were not relevant for cell attachment because PEG6000 from Merck, with larger polymer islands, and PEG6000 from S.A., with very small islands, led both to cell attachment and 2D growth. Moreover, we observed that there were strong variations in the island size in different areas of the same dish (S6 Fig). Therefore, we concluded that the surface was not responsible for the spheroidal growth of the cells.

**Fig 6:**
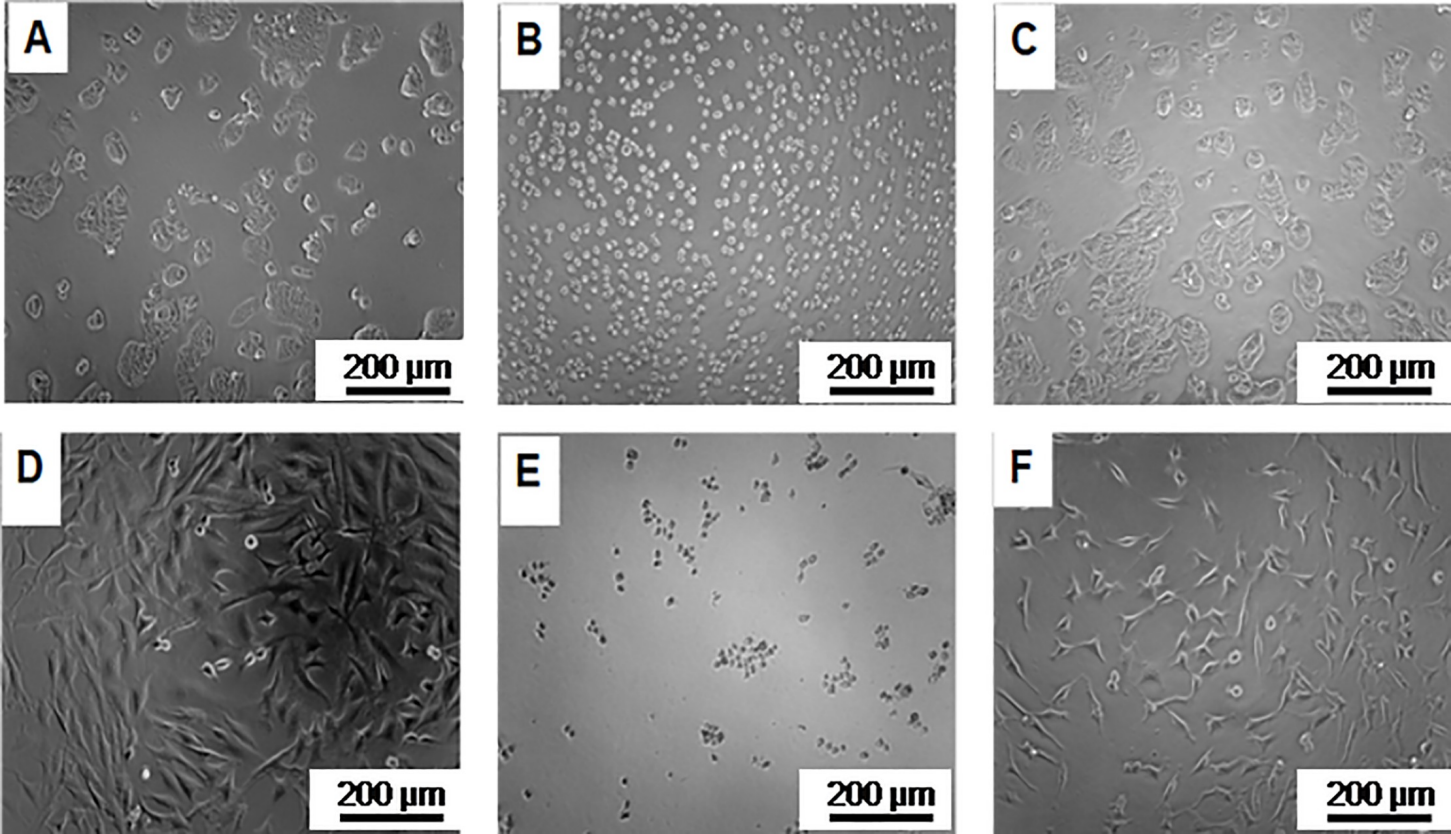
Micrographs of HT-29 (A-C) and Hela (D-F) cells without treatment (A, D), or incubated for 5 mins in PEG6000 from C.E. (B, E) and S.A. (C, F) and imaged 48 h after seeding. The scale bars indicate 200 μm.

Finally, we checked if a direct interaction between cells and polymer could be responsible for the difference in cell attachment, since after incubation with the polymer no further rinsing of the culture dish was performed before the cell suspension was added to the wells. Therefore, we incubated the cells in 3% PEG6000 from C.E and from S.A. and seeded them on an uncoated 96-well plate. In Fig 6, cells were imaged 48 h after seeding.

The micrographs in Fig 6 show that both cell lines, HT-29 and HeLa, gave rounded single cells when pre-incubated in 3% PEG6000 from C.E. for 5 mins before seeding in the cell culture plate. In contrast, untreated control cells and PEG6000 (S.A.) incubated cells showed 2D growth. This result suggests that the spheroidal growth observed for cells incubated in PEG6000 from C.E. was due to a direct cell-polymer interaction rather than to the effect of the surface coating.

## Discussion

In the present study, we tested the cell repellent properties of surfaces coated with PEG6000 from different distributors upon simple incubation. While we found no significant differences in the chemical profile of the different materials except a small additional peak at 4000 Da in the MALDI spectrum of PEG6000 from (C.E.), we observed a significant difference in the biological response in terms of cell growth. In order to understand the difference in cell behavior, we first studied the surface coverage of the cell culture dishes with the different PEG6000s. The differences between the surface coatings using the different PEG6000s were not significantly related to the observed cell behavior. We therefore checked whether the direct interaction between PEG and cells could be responsible for the cell behavior. Indeed, when we tested cell growth after short-term incubation with PEG6000 from C.E. and from S.A., we observed that already the pre-incubated cells showed differences in morphology. Cells incubated in C.E. PEG6000, growing later in spheroids, were already more roundish as compared to the cells incubated in S.A. PEG6000. To our knowledge this is the first study performed on PEG6000 showing drastic biological differences in absence of significantly different chemical or physical properties of the polymeric molecules.

The variations of biological entities in response to similar PEGs from different distributors can be of scientific relevance because this polymer is broadly used in pharmaceutics, as treatment for chronic constipation [21,22], in preparation for colonoscopy or gut imaging procedures [23] or even as protection against colon cancer in humans [22] as well as for development of nanodrugs [24,25]. PEG molecules are known to interact in different ways with cell surfaces.

The interaction of higher molecular weight PEG, such as 8000 and above, with the intestinal cells increases cell proliferation *in vitro* [26] and improves the gut barrier function in animal experiments [27–29].

McNamee *et al*. [30] reported that malignant melanoma B16F10 cells showed strong differences in their morphology due to binding to a PEG brush, depending on the number oxyethylene (OE) groups and headgroups present on the PEG chain. Upon attachment of melanoma cells onto the glass surface modified with PEG113 (MW: 5000 Da similar to the PEG6000 used in the present study), cell morphology became roundish comparable to the one as we observed with PEG6000 from C.E. (Fig 6B). However, it has to be noted that the cell lines used in our study and in those used in the study by McNamee *et al*.[30] are different. Moreover, the cells in the experimental setup of McNamee *et al*.[30] interact with a polymer brush and therefore cannot be completely enwrapped by the polymer, while in our case the polymer is free in solution. In general, it has to be stated that higher molecular weight polymers tend to partially coil [31]. The coiling and compaction effects are more pronounced in PEG6000 from C.E. (higher melting temperature and smaller hydrodynamic radius) than in PEG6000s from other distributors, even if they are slightly longer than the polymer from C.E..

This study showed that small differences in the composition of polymers can have a drastic impact on the behavior of biological entities. Our research should raise awareness about the validity of toxicity data as well as of drug delivery data when the same polymer from different distributors is used.

## Supporting information

S1 Fig **FTIR spectra of PEG6000 from Sigma-Aldrich (red) and Carlo Erba (blue).** Overlay (lower panel, left) and magnification (lower panel, right) of both spectra to visualize the small differences.
(DOC)

S2 Fig ^1^H-NMR measurement of PEG6000 from (A) S.A.; (B) C.E.
(DOC)

S3 Fig Q-TOF-MS spectra measured for PEG6000 from A) C.E., B) Merck, C) S.A., and D) Acros.
(DOC)

S4 Fig Triple chromatogram of PEG6000 from (A) S.A., C.E., and C) Merck: The red diagram represents the data measured for the refractive index; the blue diagram depicts the viscosity measurement and the green one the RALS data.
(DOC)

S5 Fig DSC spectrum of PEG6000 from Merck (black), C.E. (red), S.A. (blue), and Acros (green).
(DOC)

S6 Fig AFM micrographs of 3 different zones of the same dish after deposition of PEG6000 from C.E. (upper panel), and Merck (lower panel).
(DOC)
