# Supplementary material for: Reproducibility warning: The curious case of polyethylene glycol 6000 and spheroid cell culture
Source: PLoS One. 2020 Mar 19;15(3):e0224002. doi: 10.1371/journal.pone.0224002 (PMC7082040; doi:10.1371/journal.pone.0224002)
Supplement: S1 Fig — FTIR spectra of PEG6000 from Sigma-Aldrich (red) and Carlo Erba (blue). Overlay (lower panel, left) and magnification (lower panel, right) of both spectra to visualize the small differences. (DOC) [file pone.0224002.s001.doc]

**Figure S1**. FTIR spectra of PEG6000 from Sigma-Aldrich (red) and Carlo Erba (blue). Overlay (lower panel, left) and magnification (lower panel, right) of both spectra to visualize the small differences.
