# Supplementary material for: Reproducibility warning: The curious case of polyethylene glycol 6000 and spheroid cell culture
Source: PLoS One. 2020 Mar 19;15(3):e0224002. doi: 10.1371/journal.pone.0224002 (PMC7082040; doi:10.1371/journal.pone.0224002)
Supplement: S2 Fig — 1H-NMR measurement of PEG6000 from (A) S.A.; (B) C.E. (DOC) [file pone.0224002.s002.doc]

**Figure S2**: ^1^H-NMR measurement of PEG6000 from (A) S.A.; (B) C.E.
