# Supplementary figures and images for: Reproducibility warning: The curious case of polyethylene glycol 6000 and spheroid cell culture
Source: PLoS One. 2020 Mar 19;15(3):e0224002. doi: 10.1371/journal.pone.0224002 (PMC7082040; doi:10.1371/journal.pone.0224002)

**A**


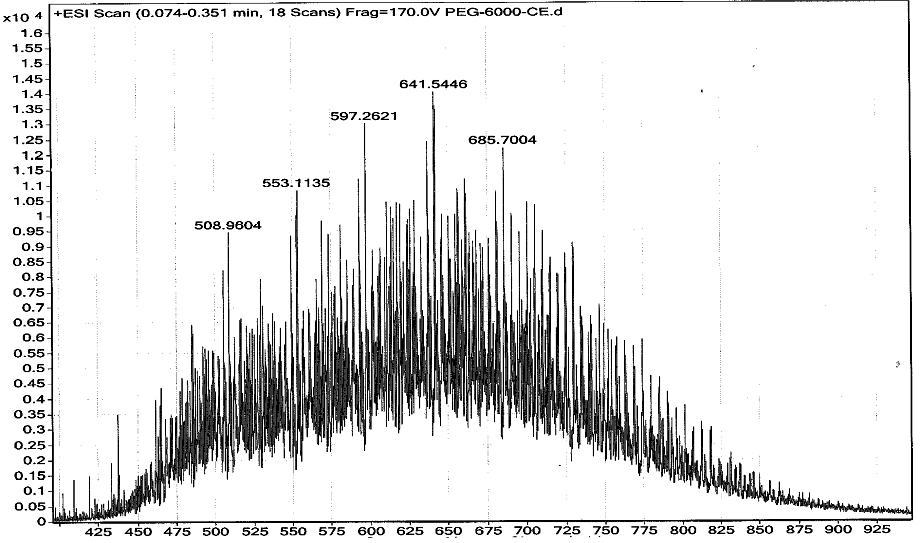


**B**


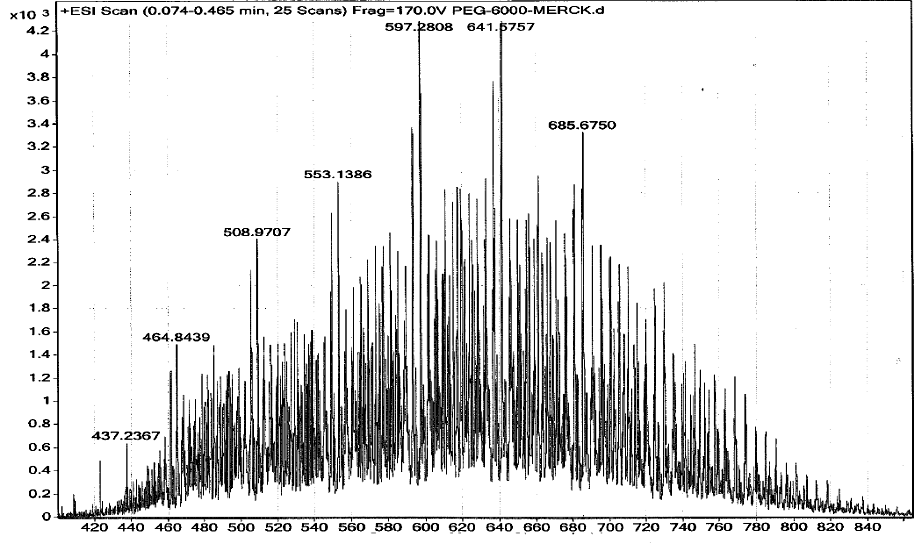


**C**


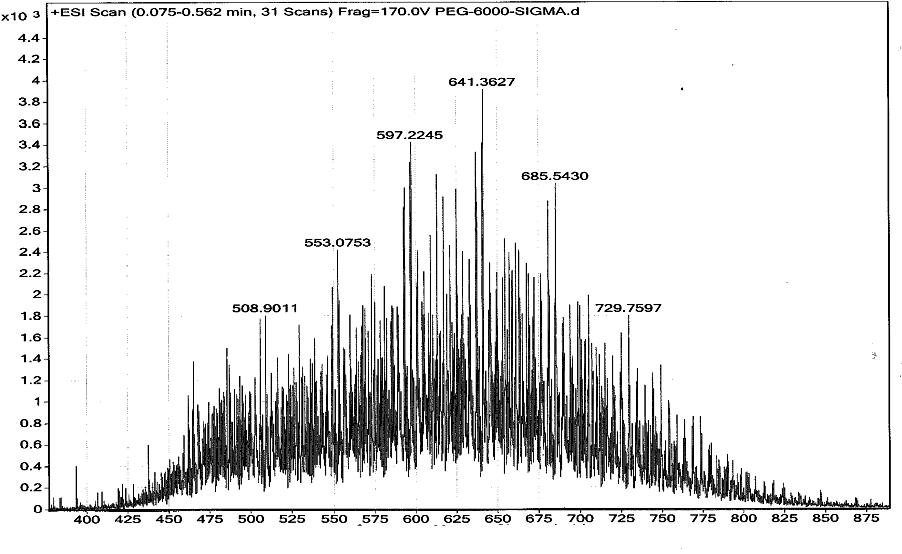


**D**


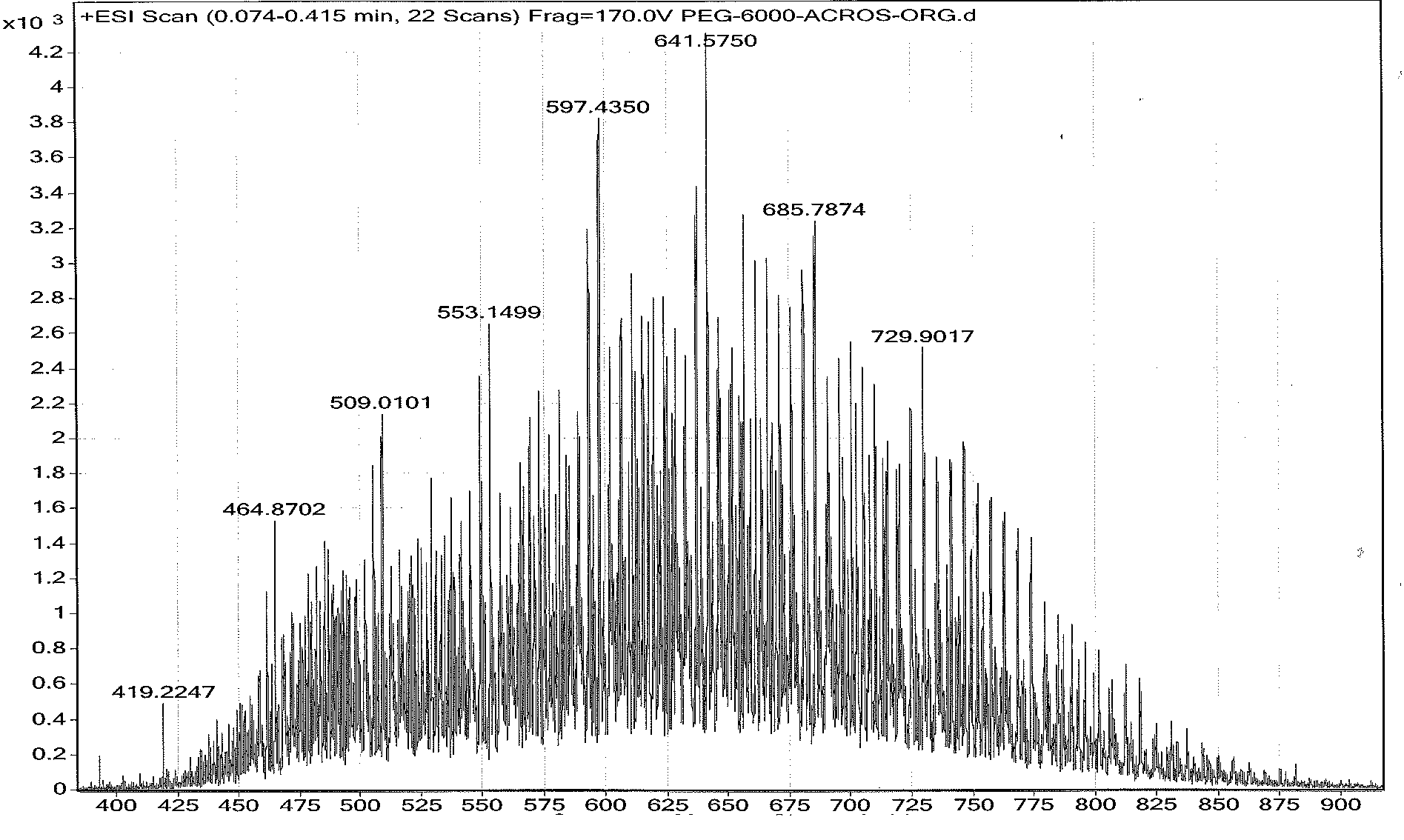


**Figure S3.** Q-TOF-MS spectra measured for PEG6000 from A) C.E., B) Merck, C) S.A., and D) Acros.

Supplement: S3 Fig — Q-TOF-MS spectra measured for PEG6000 from A) C.E., B) Merck, C) S.A., and D) Acros. (DOC) [file pone.0224002.s003.doc]
