# Supplementary material for: Reproducibility warning: The curious case of polyethylene glycol 6000 and spheroid cell culture
Source: PLoS One. 2020 Mar 19;15(3):e0224002. doi: 10.1371/journal.pone.0224002 (PMC7082040; doi:10.1371/journal.pone.0224002)
Supplement: S4 Fig — Triple chromatogram of PEG6000 from (A) S.A., C.E., and C) Merck: The red diagram represents the data measured for the refractive index; the blue diagram depicts the viscosity measurement and the green one the RALS data. (DOC) [file pone.0224002.s004.doc]

**Figure S4**. Triple chromatogram of PEG6000 from (A) S.A., C.E., and C) Merck: The red diagram represents the data measured for the refractive index; the blue diagram depicts the viscosity measurement and the green one the RALS data.
