# Supplementary material for: Reproducibility warning: The curious case of polyethylene glycol 6000 and spheroid cell culture
Source: PLoS One. 2020 Mar 19;15(3):e0224002. doi: 10.1371/journal.pone.0224002 (PMC7082040; doi:10.1371/journal.pone.0224002)
Supplement: S5 Fig — DSC spectrum of PEG6000 from Merck (black), C.E. (red), S.A. (blue), and Acros (green). (DOC) [file pone.0224002.s005.doc]

**Figure S5.** DSC spectrum of PEG6000 from Merck (black), C.E. (red), S.A. (blue), and Acros (green)
