# Supplementary material for: Reproducibility warning: The curious case of polyethylene glycol 6000 and spheroid cell culture
Source: PLoS One. 2020 Mar 19;15(3):e0224002. doi: 10.1371/journal.pone.0224002 (PMC7082040; doi:10.1371/journal.pone.0224002)
Supplement: S6 Fig — AFM micrographs of 3 different zones of the same dish after deposition of PEG6000 from C.E. (upper panel), and Merck (lower panel). (DOC) [file pone.0224002.s006.doc]

**Figure S6.** AFM micrographs of 3 different zones of the same dish after deposition of PEG6000 from C.E. (upper panel), and Merck (lower panel).
